# Multimarker approach for the prediction of microvascular obstruction after acute ST-segment elevation myocardial infarction: a prospective, observational study

**DOI:** 10.1186/s12872-016-0415-z

**Published:** 2016-11-28

**Authors:** Hans-Josef Feistritzer, Sebastian Johannes Reinstadler, Gert Klug, Martin Reindl, Sebastian Wöhrer, Christoph Brenner, Agnes Mayr, Johannes Mair, Bernhard Metzler

**Affiliations:** 1 University Clinic of Internal Medicine III, Cardiology and Angiology, Medical University of Innsbruck, Anichstraße 35, A-6020 Innsbruck, Austria; 2 Department of Radiology, Medical University of Innsbruck, Anichstraße 35, A-6020 Innsbruck, Austria

**Keywords:** Myocardial infarction, Biomarker, Cardiovascular magnetic resonance imaging

## Abstract

**Background:**

Presence of microvascular obstruction (MVO) derived from cardiac magnetic resonance (CMR) imaging is among the strongest outcome predictors after ST-segment elevation myocardial infarction (STEMI). We aimed to investigate the comparative predictive values of different biomarkers for the occurrence of MVO in a large cohort of reperfused STEMI patients.

**Methods:**

This study included 128 STEMI patients. CMR imaging was performed within the first week after infarction to assess infarct characteristics, including MVO. Admission and peak concentrations of high-sensitivity cardiac troponin T (hs-cTnT), creatine kinase (CK), N-terminal pro-B-type natriuretic peptide (NT-proBNP), high-sensitivity C-reactive protein (hs-CRP), lactate dehydrogenase (LDH), aspartate transaminase (AST) and alanine transaminase (ALT) were measured.

**Results:**

MVO was detected in 69 patients (54%). hs-cTnT, CK, hs-CRP, LDH, AST and ALT peak concentrations showed similar prognostic value for the prediction of MVO (area under the curve (AUC) = 0.77, 0.77, 0.68, 0.79, 0.78 and 0.73, all *p* > 0.05), whereas the prognostic utility of NT-proBNP was weakly lower (AUC = 0.64, *p* < 0.05). Combination of these biomarkers did not increase predictive utility compared to hs-cTnT alone (*p* = 0.349).

**Conclusions:**

hs-cTnT, CK, hs-CRP, LDH, AST and ALT peak concentrations provided similar prognostic value for the prediction of MVO. The prognostic utility of NT-proBNP was lower. Combining these biomarkers could not further improve predictive utility compared to hs-cTnT alone.

## Background

The broad implementation of early primary percutaneous coronary intervention (PPCI) for acute ST-segment elevation myocardial infarction (STEMI) resulted in a significant improvement of clinical outcomes [1, 2]. Nevertheless, despite restoration of epicardial blood flow, adequate myocardial reperfusion cannot be achieved in a significant portion of patients-a phenomenon called ‘no reflow’ [3]. Three distinct pathophysiological processes are critical in the development of ‘no reflow’ after PPCI for STEMI: distal embolization, ischemia-reperfusion injury, and individual susceptibility [4].

Today cardiac magnetic resonance (CMR) imaging allows a comprehensive infarct characterization including the assessment of microvascular injury [5]. Microvascular injury detected by the use of CMR imaging is generally called ‘microvascular obstruction’ (MVO) [6]. In the so far largest prospective, multicenter study comprising more than 700 STEMI patients MVO provided independent and incremental prognostic value for the occurrence of major adverse cardiac events within 1 year after infarction [7]. In the study by Cochet et al. MVO provided 84% sensitivity and 65% specificity for the prediction of major adverse cardiac events 1 year after reperfused AMI [8]. Another study in STEMI patients proved MVO as an independent outcome predictor during a median long-term follow-up of 52 months [9]. Importantly, presence of MVO provides incremental information over traditional outcome parameters like left ventricular (LV) ejection fraction and infarct size (IS) [3, 10]. Identification of MVO, therefore, could allow for ideal risk stratification in the early stage after acute STEMI, but is still hampered due to the limited availability of CMR in clinical routine [11]. A biomarker model for prediction of MVO, which could easily be applied in a broad range of STEMI patients, might provide a practicable and cost effective alternative to CMR.

An association between serially measured cardiac troponin concentrations and CMR derived MVO has previously been reported for this patient cohort [12, 13]. However, these studies were hampered either due to a small sample size or the use of non-high-sensitivity troponin assays. Furthermore, N-terminal pro-B-type natriuretic peptide (NT-proBNP) levels obtained upon hospital admission for acute STEMI might be predictive for the presence of MVO [14]. However, limited data are available regarding the relation of CMR derived MVO and other clinically available biomarkers apart from cardiac troponin and NT-proBNP [12–14]. Therefore, the aims of the present study were 1) to investigate the association of CMR-determined MVO with admission and peak concentrations of routinely measured laboratory markers (high-sensitivity cardiac troponin T (hs-cTnT), creatine kinase (CK), NT-proBNP, high-sensitivity C-reactive protein (hs-CRP), lactate dehydrogenase (LDH), aspartate transaminase (AST) and alanine transaminase (ALT)), 2) to assess the prognostic value of these biomarkers for the prediction of MVO and 3) to analyze the prognostic utility of a combined biomarker panel.

## Methods

### Study population

One hundred and twenty-eight patients with first STEMI admitted to our coronary care unit were recruited to this single-center, prospective, observational study. Diagnosis of STEMI was based on the redefined ESC/ACC committee criteria [15]. Inclusion criteria were reperfusion by PPCI and no contraindication for CMR examination. Exclusion criteria were age below 18 years, an estimated glomerular filtration rate < 30 ml/min/1.73 m^2^ and Killip class ≥ 3 at admission. Data on patient characteristics were acquired with the help of a standardized questionnaire during hospitalization. Ischemia time was defined as the delay from symptom onset to the time-point of first balloon-inflation. The study complies with the ethical guidelines of the 1975 Declaration of Helsinki and was approved by the local ethics committee of Medical University of Innsbruck. Written informed consent was obtained from all patients before inclusion into the study.

### Biochemical analysis

Blood samples were taken from a peripheral vein and immediately analyzed at our central laboratory. High-sensitivity cardiac troponin T (hs-cTnT) concentrations were measured using a fifth-generation high-sensitivity assay (Roche Diagnostics, Mannheim, Germany) [16, 17]. The analytical limit of detection was 5 ng/l and the 99^th^ percentile upper reference limit was 14 ng/l. The 10% coefficient of variation was 13 ng/l. Plasma NT-proBNP concentrations were measured as described in detail previously [18, 19]. The analytical limit of detection of NT-proBNP was 5 ng/l. Creatine kinase (CK), high-sensitivity C-reactive protein (hs-CRP), lactate dehydrogenase (LDH), aspartate transaminase (AST), and alanine transaminase (ALT) activities were measured by routine assays as described previously [16, 18].

hs-cTnT concentrations were determined on admission, subsequently three times during the first 24 h and then daily until day 4 or discharge. All other biomarkers were measured on admission and subsequently once daily up to day 4 after PPCI or discharge. Biomarker peak concentrations are regarded as the highest values in the concentration time-course.

### Cardiac magnetic resonance imaging

All CMR scans were performed on a 1.5 Tesla Magnetom Avanto scanner (Siemens, Erlangen, Germany). The imaging protocol has been described in detail previously [12]. In brief, true fast imaging steady-state precession (true-FISP) bright-blood sequences in the LV short axis were acquired to assess LV function and morphology. Image post-processing was performed using standard software (ARGUS, Siemens). IS and presence of MVO were derived from late gadolinium-enhanced images as previously described [9]. A threshold of + 5 standard deviations was defined as ‘hyperenhancement’ [20]. MVO was defined as a persisting area of hypoenhancement within hyperenhanced myocardium [9].

### Statistical analysis

Statistical analysis was performed using SPSS Statistics 22.0.0 (IBM, Armonk, NY, USA) and MedCalc 15.8 (Ostend, Belgium). To test for normal distribution (ND), Shapiro-Wilk test was used. Continuous data are expressed as mean ± standard deviation (SD) or as median with interquartile range (IQR) as appropriate. Categorical data are expressed as numbers with corresponding percentages. Pearson’s (if ND) or Spearman’s rank (if not ND) correlation coefficients were calculated. Differences in continuous variables between groups were calculated by Student’s t-test, if ND. Otherwise, Mann-Whitney U test was used. To test for group-differences of categorical variables χ^2^-test was applied.

Receiver operator characteristics (ROC) analysis was used to determine the predictive value (area under the curve (AUC)) of biomarkers. Biomarkers which significantly differed between patients with and without MVO were included into ROC analyses. Binary logistic regression analysis was used as a statistical tool to combine biomarkers as previously described [17]. The potentially incremental information of a combined biomarker model for the prediction of MVO was assessed by *c*-statistics. *C*-statistic results were compared using the method previously described by DeLong et al [21]. Two-tailed p-values < 0.05 were considered statistically significant.

## Results

### Patients and infarct-related characteristics

Mean age of the study cohort was 58 ± 10 years; 12 patients (9%) were female. Baseline characteristics of the study cohort are shown in Table 1. The median delay to reperfusion was 220 min (IQR 155–417 min). Sixty-one patients (48%) presented with anterior infarct location. Seventy (56%), 34 (27%) and 21 (17%) patients showed 1-, 2- and 3-vessel disease according to coronary angiography, respectively. Culprit only PPCI was performed in all 128 patients. The culprit vessel was the right coronary artery in 55 (43%), the left anterior descending artery in 60 (47%), the left circumflex artery in 12 (9%) and the ramus intermedius in 1 (1%) cases.

**Table 1:** Patient characteristic of the overall cohort (*n =* 128) and after stratification for presence of MVO

Patient characteristics	Overall cohort	Presence of MVO		
	(*n* = 128)	no (*n* = 59, 46%)	yes (*n* = 69, 54%)	*p*
Age, years	58 ± 10	57 ± 10	58 ± 10	0.925
Female, n (%)	12 (9)	8 (14)	4 (6)	0.223
Body mass index, kg/m^2^	26 (25–30)	27 (26–29)	26 (24–30)	0.522
Family history for AMI, n (%)	34 (27)	19 (32)	15 (22)	0.229
Current smokers, n (%)	63 (49)	28 (48)	35 (51)	0.727
RR_sys_, mmHg	125 ± 24	126 ± 23	123 ± 24	0.491
RR_dia_, mmHg	77 ± 15	78 ± 14	76 ± 15	0.309
Leucocyte count admission, G/l	11.7 (8.8–14.5)	11.5 (8.5–14.5)	12.0 (9.0–14.6)	0.649
Leucocyte count max, G/l	12.7 (10.8–15.8)	11.9 (10.3–15.2)	13.1 (11.7–16.4)	0.061
Total cholesterol, mg/dl	192 ± 44	185 ± 41	198 ± 47	0.108
Low-density lipoprotein, mg/dl	128 ± 42	123 ± 39	132 ± 44	0.230
High-density lipoprotein, mg/dl	42 (36–49)	40 (33–53)	43 (38–49)	0.320
Triglycerides, mg/dl	102 (78–151)	108 (80–163)	96 (77–142)	0.276
Plasma glucose admission, mg/dl	135 (115–161)	134 (116–163)	137 (112–160)	0.728
HbA1c, %	5.6 (5.4–6.0)	5.6 (5.4–6.1)	5.6 (5.3–5.9)	0.792
Creatinine admission, mg/dl	0.93 (0.82–1.05)	0.92 (0.76–1.07)	0.93 (0.83–1.04)	0.877
Creatinine max, mg/dl	1.04 (0.92–1.15)	1.03 (0.91–1.13)	1.06 (0.96–1.20)	0.261
hs-cTnT admission, ng/l	226 (29–2168)	201 (18–1388)	523 (34–4001)	**0.036**
hs-cTnT max, ng/l	5254 (2169–8735)	2866 (1026–5524)	6985 (4193–13639)	**<0.001**
CK admission, U/l	353 (174–1182)	301 (148–862)	435 (207–2433)	**0.048**
CK max, U/l	2111 (1168–3674)	1228 (625–2323)	2776 (1749–4686)	**<0.001**
NT-proBNP admission, ng/l	139 (66–511)	128 (66–360)	159 (71–687)	0.487
NT-proBNP max, ng/l	717 (184–1700)	360 (131–924)	1084 (312–2649)	**0.003**
hs-CRP admission, mg/dl	0.23 (0.11–0.61)	0.34 (0.15–0.66)	0.19 (0.09–0.56)	0.119
hs-CRP max, mg/dl	2.20 (0.96–4.56)	1.53 (0.64 3.02)	2.84 (1.68–7.44)	**<0.001**
LDH admission, U/l	238 (196–364)	222 (182–301)	257 (207–493)	**0.009**
LDH max, U/l	593 (354–882)	358 (271–624)	742 (534–1182)	**<0.001**
AST admission, U/l	82 (34–210)	57 (31–113)	126 (40–306)	**0.008**
AST max, U/l	239 (128–423)	151 (75–256)	340 (213–547)	**<0.001**
ALT admission, U/l	37 (27–58)	31 (25–46)	45 (32–75)	**0.003**
ALT max, U/l	60 (42–87)	47 (32–69)	71 (52–111)	**<0.001**
γGT admission, U/l	37 (25–52)	36 (26–50)	39 (23–59)	0.857
γGT max, U/l	40 (26–63)	37 (26–51)	41 (26–71)	0.482
AP admission, U/l	64 (53–79)	64 (52–80)	63 (54–76)	0.918
AP max, U/l	64 (53–81)	64 (52–83)	64 (54–81)	0.951
Total bilirubin admission, mg/dl	0.49 (0.38–0.69)	0.44 (0.37–0.65)	0.52 (0.40–0.73)	0.160
Total bilirubin max, mg/dl	0.62 (0.43–0.84)	0.60 (0.42–0.84)	0.62 (0.45–0.85)	0.668

*MVO* Microvascular Obstruction, *AMI* Acute Myocardial Infarction, *RR*_*sys*_ Systolic Blood Pressure, *RR*_*dia*_ Diastolic Blood Pressure, *hs-cTnT* High-Sensitivity Cardiac Troponin T, *CK* Creatine Kinase, *NT-proBNP* N-terminal pro-B-type natriuretic peptide, *hs-CRP* High-Sensitivity C-Reactive Protein, *LDH* Lactate Dehydrogenase, *AST* Aspartate Transaminase, *ALT* Alanine Transaminase, *γGT* Gamma-Glutamyltransferase, *AP* Alkaline Phosphatase

Bold data indicate statistical significance

CMR scans were performed at a median of 3 days after infarction (IQR 2–4 days). Median IS was 13% (IQR 8–25%). Mean LVEF was 54 ± 10%. MVO was present in 69 patients (54%).

### Predictors of MVO

The delay from symptom onset to mechanical reperfusion did not significantly differ between patients with and without MVO (median = 213 min, IQR 149–399 min vs median = 240 min, IQR 154–424 min; *p* = 0.469). Patients showing MVO were more likely to have anterior infarcts compared to patients without MVO (*n* = 39, 57% vs *n =* 22, 37%; *p* = 0.030). No association was detected between the presence of MVO and the number of diseased epicardial coronary arteries (*p* = 0.480).

Patients with presence of MVO showed significantly higher IS (media*n =* 21%, IQR 14–30% vs median = 9%, IQR 3–13%; *p* < 0.001), EDV (153 ± 30 ml vs 140 ± 26 ml; *p* = 0.010), ESV (median = 73 ml, IQR 60–90 ml vs median = 56 ml, IQR 44–72 ml; *p* < 0.001) and lower LVEF (50 ± 9% vs 59 ± 10%; *p* < 0.001). The occurrence of MVO did not significantly differ between patients with one- and multivessel disease (*p* = 0.286).

**Fig. 1:**
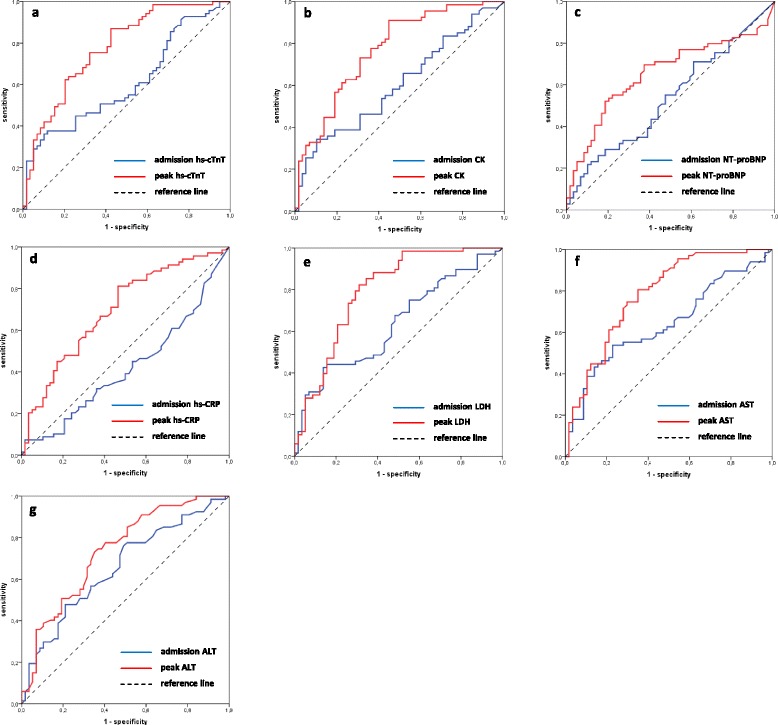
Receiver operator characteristics (ROC) analyses comparing the predictive utility of admission and peak concentrations of **a**) hs-cTnT (AUC = 0.61, 95% CI 0.52–0.69 vs AUC = 0.77, 95% CI 0.68–0.85; *p* < 0.001), **b**) CK (AUC = 0.60, 95% CI 0.51–0.69 vs AUC = 0.77, 95% CI 0.69–0.84; *p* = 0.001), **c**) NT-proBNP (AUC = 0.54, 95% CI 0.45–0.62 vs AUC = 0.64, 95% CI 0.55–0.73; *p* = 0.011) **d**) hs-CRP (AUC = 0.58, 95% CI 0.49–0.67 vs AUC = 0.68, 95% CI 0.60–0.76; *p* = NA), **e**) LDH (AUC = 0.64, 95% CI 0.54–0.72 vs AUC = 0.79, 95% CI 0.71–0.86; *p* = 0.003), **f**) AST (AUC = 0.64, 95% CI 0.55–0.72 vs AUC = 0.78, 95% CI 0.70–0.85, *p* = 0.005) and **g**) ALT (AUC = 0.66, 95% CI 0.57–0.74 vs AUC = 0.73, 95% CI 0.64–0.80; *p* = 0.049). hs-cTnT = High-Sensitivity Cardiac Troponin T; CK = Creatine Kinase; NT-proBNP = N-terminal pro-B-type natriuretic peptide; hs-CRP = High-Sensitivity C-Reactive Protein; LDH = Lactate Dehydrogenase; AST = Aspartate Transaminase; ALT = Alanine Transaminase

Differences in biomarker levels between patients with and without MVO are shown in Table 1.

hs-cTnT concentrations at admission provided an AUC value of 0.61 (95% CI 0.52–0.69) for the prediction of MVO (Fig. 1a). AUCs did not significantly differ between admission hs-cTnT, CK, NT-proBNP, hs-CRP, LDH, AST and ALT (range 0.54, 95% CI 0.45–0.62 to 0.66, 95% CI 0.57–0.74; all *p* > 0.080).

Biomarker peak concentrations provided significantly higher prognostic value for the prediction of MVO than corresponding admission values (all *p* < 0.050) (Fig. 1).

Peak hs-cTnT concentrations provided an AUC of 0.77 (95% CI 0.68–0.85) for the prediction of MVO. Peak CK (AUC = 0.77, 95% CI 0.69–0.84; *p* = 0.882), hs-CRP (AUC = 0.68, 95% CI 0.60–0.76; *p* = 0.108), LDH (AUC = 0.79, 95% CI 0.71–0.86; *p* = 0.336), AST (AUC = 0.78, 95% CI 0.70–0.85; *p* = 0.517) and ALT (AUC = 0.73, 95% CI 0.64–0.80; *p* = 0.312) showed similar AUCs for the prediction of MVO compared to hs-cTnT. NT-proBNP peak concentrations (AUC = 0.64, 95% CI 0.55–0.73) exhibited significantly lower predictive utility compared to peak hs-cTnT (*p* = 0.027), CK (*p* = 0.031), LDH (*p* = 0.007) and AST (*p* = 0.016) concentrations. Optimal biomarker cut-off values with corresponding sensitivity, specificity as well as positive and negative predictive values for the prediction of MVO are summarized in Table 2.

**Table 2:** Biomarker cut-off values providing optimal sensitivity, specificity as well as positive and negative predictive values

	optimal cut-off	sensitivity	specificity	PPV	NPV
hs-cTnT max, ng/l	4387	75	68	73	70
CK max, U/l	1959	73	69	73	69
NT-proBNP max, ng/l	551	70	63	69	64
hs-CRP max, mg/dl	2,11	67	63	68	62
LDH max, U/l	496	82	70	76	77
AST max, U/l	224	75	71	75	71
ALT max, U/l	57	73	64	70	67

*hs-cTnT* High-Sensitivity Cardiac Troponin T, *CK* Creatine Kinase, *NT-proBNP* N-terminal pro-B-type natriuretic peptide, *hs-CRP* High-Sensitivity C-Reactive Protein, *LDH* Lactate Dehydrogenase, *AST* Aspartate Transaminase, *ALT* Alanine Transaminase

**Fig. 2:**
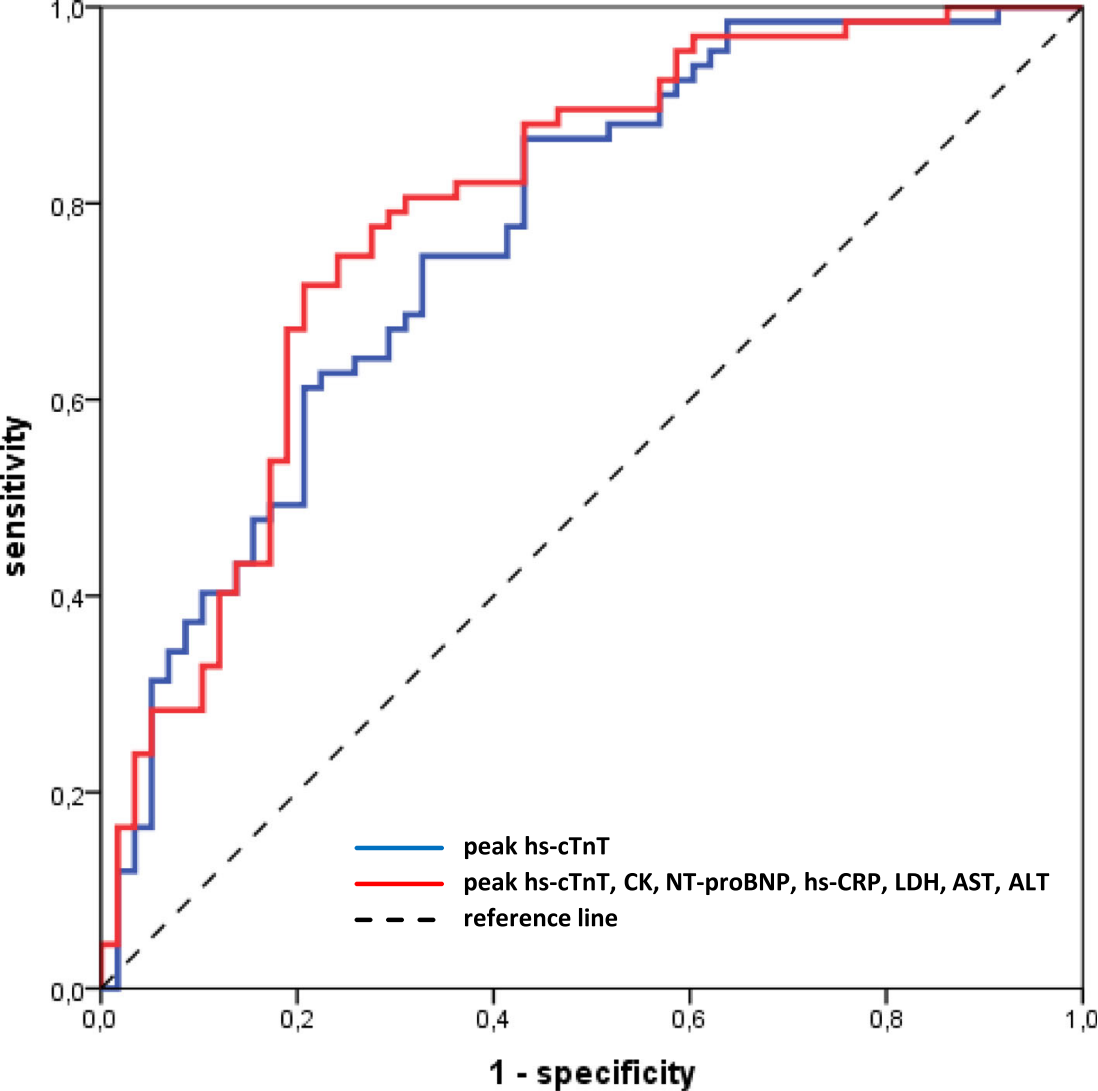
Receiver operator characteristics (ROC) curves of peak hs-cTnT concentrations and a combined biomarker model. Combination of biomarkers did not result in significantly higher prognostic value for the prediction of MVO compared to hs-cTnT alone (AUC = 0.79, 95% CI 0.71–0.87 vs AUC = 0.77, 95% CI 0.68–0.85; *p* = 0.349). hs-cTnT = High-Sensitivity Cardiac Troponin T; CK = Creatine Kinase; NT-proBNP = N-terminal pro-B-type natriuretic peptide; hs-CRP = High-Sensitivity C-Reactive Protein; LDH = Lactate Dehydrogenase; AST = Aspartate Transaminase; ALT = Alanine Transaminase

Including peak CK, NT-proBNP, hs-CRP, LDH, AST and ALT concentrations additionally to peak hs-cTnT levels did not result in a significantly higher accuracy for the prediction of MVO (AUC = 0.79, 95% CI 0.71–0.87 vs AUC = 0.77, 95% CI 0.68–0.85; *p* = 0.349) (Fig. 2).

## Discussion

This study for the first time comprehensively assessed the incremental value of routine biomarkers for the prediction of MVO after reperfused STEMI. The major findings were that 1) presence of MVO was associated with higher plasma concentrations of hs-cTnT, CK, hs-CRP, LDH, AST and ALT; 2) biomarker peak concentrations provided significantly higher prognostic value compared to admission values; 3) peak concentrations of hs-cTnT, CK, hs-CRP, LDH, AST and ALT showed similar prognostic value for the prediction of MVO, whereas the prognostic utility of peak NT-proBNP was lower; and 4) a model including peak CK, NT-proBNP, hs-CRP, LDH, AST and ALT concentrations additionally to hs-cTnT did not result in a significantly higher accuracy for the prediction of MVO.

Inadequate myocardial reperfusion despite restoration of epicardial coronary artery patency is attributed to microvascular injury [22]. As previously shown, application of modern antiplatelet therapy partly improves microvascular perfusion [23]. Interestingly, removal of thrombotic material by thrombus aspiration is ineffective in improvement of myocardial reperfusion [24]. In the present study microvascular injury, defined as the presence or absence of CMR derived MVO, was detected in 54% of patients. This is in line with data from literature, reporting an up to 60% rate of MVO in patients receiving primary PCI for acute STEMI [3]. In the present study culprit only revascularization was performed during PPCI. Thus, the impact of culprit only versus complete PPCI on MVO could not be analysed in the present study. This important topic was recently investigated in a well-conducted meta-analysis [25].

Besides its ability for accurate quantification of ventricular function, morphology and infarct size, CMR imaging has emerged as the most reliable imaging modality to detect microvascular injury and is therefore more and more used to define surrogate endpoints in clinical trials [5, 26, 27]. There is strong evidence that presence of MVO derived from late gadolinium-enhanced images is the best CMR prognosticator regarding clinical outcome after acute reperfused STEMI [3, 7]. Nevertheless, its determination is hampered since CMR is still a rarely available, expensive tool with restricted application in clinical routine. Thus, implementation of a biomarker model, which reliably allows for the prediction of MVO, is of clinical and prognostic importance [28].

In the present study, time to reperfusion did not differ between patients with and without MVO. Presumably, this is due to the fact that microvascular dysfunction might persist even after restoration of epicardial blood flow [3, 29].

In line with data from literature, we detected a relation between the presence of MVO and infarct size, LV systolic function and morphology [30–32].

An association between cardiac troponin concentrations and CMR derived MVO has already been reported by several studies [13, 33, 34]. Notably, the significance of these studies is limited due to an either small sample size and poorly defined patient selection (STEMI and non-STEMI patients included) or inconsistent reperfusion strategies. These limitations were obviated in the study by Mayr et al, since the relation between MVO and cardiac troponin levels was confirmed in a large, well-defined cohort of STEMI patients [12]. However, in this study a non-high-sensitivity cardiac troponin T assay was used. These assays provide lower prognostic value and are associated with a longer troponin-blind period compared to new-generation high-sensitivity assays [35]. This fact might particularly impact on the prognostic utility of admission troponin levels.

The present study extends previous findings, as we investigated the predictive value of a broad range of routinely used laboratory markers including high-sensitivity cardiac troponin T. All patients were exclusively reperfused by primary PCI, which is of importance since reperfusion strategy might influence the occurrence of MVO. Furthermore, high patient selection, as performed in the present study, reduced the presence of co-morbidities and consequently allowed to investigate predictors of MVO independently of concomitant cardiovascular disease.

Interestingly, peak concentrations of hs-cTnT, CK, hs-CRP, LDH, AST and ALT provided similar prognostic values for the prediction of MVO. This might be of practical significance, in particular if high-sensitivity troponin assays are not available in clinical routine. On the other hand, combining hs-cTnT with other biomarkers did not further improve the prognostic value compared to a model solely including hs-cTnT.

To a certain extent, biomarkers investigated in the present study have already been linked to clinical outcome following AMI [36]. Moreover, in AMI patients prognostic utility was even proved for novel, upcoming biomarkers like galectin-3 [37]. Although an association between biomarkers and hard clinical endpoints was shown in previous studies, the present study adds some causal relation, particularly since MVO is among the strongest predictors of poor outcome after STEMI [7].

Besides its prognostic value, the therapeutic relevance of MVO has been demonstrated in several studies. For instance, modern antiplatelet therapy might improve microvascular perfusion [23]. Moreover, in patients with successfully reperfused AMI treatment with statins before infarction was associated with a reduction of microvascular injury [38].

### Limitations

This is the largest CMR study investigating a comprehensive biomarker model for the prediction of MVO. Nevertheless, further confirmation in larger cohorts is necessary. Remarkably, sensitivity and specificity is less than 80% for almost all biomarkers observed in the present study. These biomarkers reflect myocardial injury, haemodynamic alterations and inflammation. However, the occurrence of MVO is a rather complex pathophysiological process and, to a certain extent, a still unresolved topic [39]. Possibly, the analysis of other, upcoming chemical markers might further improve the predictive value for the occurrence of MVO and should be investigated in future studies [36, 40].

In the present study the proportion of female patients was very low. Out of twelve female patients totally included only four patients developed MVO. Therefore, gender-specific, valid statistical analysis could not be performed. However, the impact of gender on outcome in STEMI patients has already been shown [41]. Thus, further studies are needed investigating gender-specific differences in the prediction of MVO.

The present study focused on the prediction of MVO derived from CMR. Therefore, angiographic assessment of myocardial blush grade and the relatively novel wire-based technique to measure the index of microcirculatory resistance were not performed in this study [42].

## Conclusions

hs-cTnT, CK, hs-CRP, LDH, AST and ALT peak concentrations provided similar prognostic value for the prediction of CMR derived MVO in acute STEMI patients reperfused by PPCI. In comparison, the prognostic utility of peak NT-proBNP was lower. Combination of these biomarkers did not add any additional prognostic value. Since presence of MVO is among the strongest surrogate end-points for adverse clinical outcome after acute STEMI, our findings could contribute to optimize risk stratification early after the acute event.

## Abbreviations

ALT: Alanine transaminase
AST: Aspartate transaminase
AUC: Area under the curve
CK: Creatine kinase
CMR: Cardiac magnetic resonance
EDV: End-diastolic volume
ESV: End-systolic volume
hs-CRP: High-sensitivity C-reactive protein
hs-cTnT: High-sensitivity cardiac troponin T
IQR: Interquartile range
IS: Infarct size
LDH: Lactate dehydrogenase
LV: Left ventricular
LVEF: Left ventricular ejection fraction
MVO: Microvascular obstruction
ND: Normal distribution
NT-proBNP: N-terminal pro-B-type natriuretic peptide
PPCI: Primary percutaneous coronary intervention
ROC: Receiver operator characteristics
SD: Standard deviation
STEMI: ST-segment elevation myocardial infarction
True-FISP: True fast imaging steady-state precession

## References

[1] Jernberg T, Johanson P, Held C, Svennblad B, Lindback J, Wallentin L (2011). Association between adoption of evidence-based treatment and survival for patients with ST-elevation myocardial infarction. JAMA.

[2] Gjesing A, Gislason GH, Kober L, Gustav Smith J, Christensen SB, Gustafsson F, Olsen AM, Torp-Pedersen C, Andersson C (2014). Nationwide trends in development of heart failure and mortality after first-time myocardial infarction 1997–2010: A Danish cohort study. Eur J Intern Med.

[3] van Kranenburg M, Magro M, Thiele H, de Waha S, Eitel I, Cochet A, Cottin Y, Atar D, Buser P, Wu E, Lee D, Bodi V, Klug G, Metzler B, Delewi R, Bernhardt P, Rottbauer W, Boersma E, Zijlstra F, van Geuns RJ (2014). Prognostic value of microvascular obstruction and infarct size, as measured by CMR in STEMI patients. JACC Cardiovasc Imaging.

[4] Niccoli G, Scalone G, Lerman A, Crea F (2016). Coronary microvascular obstruction in acute myocardial infarction. Eur Heart J.

[5] Klug G, Metzler B (2013). Assessing myocardial recovery following ST-segment elevation myocardial infarction: short- and long-term perspectives using cardiovascular magnetic resonance. Expert Rev Cardiovasc Ther.

[6] Schaaf MJ, Mewton N, Rioufol G, Angoulvant D, Cayla G, Delarche N, Jouve B, Guerin P, Vanzetto G, Coste P, Morel O, Roubille F, Elbaz M, Roth O, Prunier F, Cung TT, Piot C, Sanchez I, Bonnefoy-Cudraz E, Revel D, Giraud C, Croisille P, Ovize M (2016). Pre-PCI angiographic TIMI flow in the culprit coronary artery influences infarct size and microvascular obstruction in STEMI patients. J Cardiol.

[7] Eitel I, de Waha S, Wohrle J, Fuernau G, Lurz P, Pauschinger M, Desch S, Schuler G, Thiele H (2014). Comprehensive prognosis assessment by CMR imaging after ST-segment elevation myocardial infarction. J Am Coll Cardiol.

[8] Cochet AA, Lorgis L, Lalande A, Zeller M, Beer JC, Walker PM, Touzery C, Wolf JE, Brunotte F, Cottin Y (2009). Major prognostic impact of persistent microvascular obstruction as assessed by contrast-enhanced cardiac magnetic resonance in reperfused acute myocardial infarction. Eur Radiol.

[9] Klug G, Mayr A, Schenk S, Esterhammer R, Schocke M, Nocker M, Jaschke W, Pachinger O, Metzler B (2012). Prognostic value at 5 years of microvascular obstruction after acute myocardial infarction assessed by cardiovascular magnetic resonance. J Cardiovasc Magn Reson.

[10] de Waha S, Eitel I, Desch S, Fuernau G, Lurz P, Stiermaier T, Blazek S, Schuler G, Thiele H (2014). Prognosis after ST-elevation myocardial infarction: a study on cardiac magnetic resonance imaging versus clinical routine. Trials.

[11] Watanabe N, Isobe S, Okumura T, Mori H, Yamada T, Nishimura K, Miura M, Sakai S, Murohara T (2016). Relationship between QRS score and microvascular obstruction after acute anterior myocardial infarction. J Cardiol.

[12] Mayr A, Klug G, Schocke M, Trieb T, Mair J, Pedarnig K, Pachinger O, Jaschke W, Metzler B (2012). Late microvascular obstruction after acute myocardial infarction: relation with cardiac and inflammatory markers. Int J Cardiol.

[13] Pernet K, Ecarnot F, Chopard R, Seronde MF, Plastaras P, Schiele F, Meneveau N (2014). Microvascular obstruction assessed by 3-tesla magnetic resonance imaging in acute myocardial infarction is correlated with plasma troponin I levels. BMC Cardiovasc Disord.

[14] Kim MK, Chung WY, Cho YS, Choi SI, Chae IH, Choi DJ, Park YB (2011). Serum N-terminal pro-B-type natriuretic peptide levels at the time of hospital admission predict of microvascular obstructions after primary percutaneous coronary intervention for acute ST-segment elevation myocardial infarction. J Interv Cardiol.

[15] Thygesen K, Alpert JS, Jaffe AS, Simoons ML, Chaitman BR, White HD, Katus HA, Apple FS, Lindahl B, Morrow DA, Chaitman BA, Clemmensen PM, Johanson P, Hod H, Underwood R, Bax JJ, Bonow RO, Pinto F, Gibbons RJ, Fox KA, Atar D, Newby LK, Galvani M, Hamm CW, Uretsky BF, Steg PG, Wijns W, Bassand JP, Menasche P, Ravkilde J, Ohman EM, Antman EM, Wallentin LC, Armstrong PW, Januzzi JL, Nieminen MS, Gheorghiade M, Filippatos G, Luepker RV, Fortmann SP, Rosamond WD, Levy D, Wood D, Smith SC, Hu D, Lopez-Sendon JL, Robertson RM, Weaver D, Tendera M, Bove AA, Parkhomenko AN, Vasilieva EJ, Mendis S (2012). Third universal definition of myocardial infarction. Eur Heart J.

[16] Feistritzer HJ, Klug G, Reinstadler SJ, Mair J, Seidner B, Mayr A, Franz WM, Metzler B (2015). Aortic stiffness is associated with elevated high-sensitivity cardiac troponin T concentrations at a chronic stage after ST-segment elevation myocardial infarction. J Hypertens.

[17] Reinstadler SJ, Feistritzer HJ, Klug G, Mair J, Tu AM, Kofler M, Henninger B, Franz WM, Metzler B (2016). High-sensitivity troponin T for prediction of left ventricular function and infarct size one year following ST-elevation myocardial infarction. Int J Cardiol.

[18] Feistritzer HJ, Reinstadler SJ, Klug G, Kremser C, Rederlechner A, Mair J, Mueller S, Franz WM, Metzler B. N-terminal pro-B-type natriuretic peptide is associated with aortic stiffness in patients presenting with acute myocardial infarction. Eur Heart J Acute Cardiovasc Care 2015;Epub ahead of print. 10.1177/2048872615610866 26452669

[19] Mayr A, Mair J, Schocke M, Klug G, Pedarnig K, Haubner BJ, Nowosielski M, Grubinger T, Pachinger O, Metzler B (2011). Predictive value of NT-pro BNP after acute myocardial infarction: relation with acute and chronic infarct size and myocardial function. Int J Cardiol.

[20] Bondarenko O, Beek AM, Hofman MB, Kuhl HP, Twisk JW, van Dockum WG, Visser CA, van Rossum AC (2005). Standardizing the definition of hyperenhancement in the quantitative assessment of infarct size and myocardial viability using delayed contrast-enhanced CMR. J Cardiovasc Magn Reson.

[21] DeLong ER, DeLong DM, Clarke-Pearson DL (1988). Comparing the areas under two or more correlated receiver operating characteristic curves: a nonparametric approach. Biometrics.

[22] Bekkers SC, Yazdani SK, Virmani R, Waltenberger J (2010). Microvascular obstruction: underlying pathophysiology and clinical diagnosis. J Am Coll Cardiol.

[23] Montalescot G, Barragan P, Wittenberg O, Ecollan P, Elhadad S, Villain P, Boulenc JM, Morice MC, Maillard L, Pansieri M, Choussat R, Pinton P (2001). Platelet glycoprotein IIb/IIIa inhibition with coronary stenting for acute myocardial infarction. N Engl J Med.

[24] Carrick D, Oldroyd KG, McEntegart M, Haig C, Petrie MC, Eteiba H, Hood S, Owens C, Watkins S, Layland J, Lindsay M, Peat E, Rae A, Behan M, Sood A, Hillis WS, Mordi I, Mahrous A, Ahmed N, Wilson R, Lasalle L, Genereux P, Ford I, Berry C (2014). A randomized trial of deferred stenting versus immediate stenting to prevent no- or slow-reflow in acute ST-segment elevation myocardial infarction (DEFER-STEMI). J Am Coll Cardiol.

[25] Moretti C, D’Ascenzo F, Quadri G, Omede P, Montefusco A, Taha S, Cerrato E, Colaci C, Chen SL, Biondi-Zoccai G, Gaita F (2015). Management of multivessel coronary disease in STEMI patients: a systematic review and meta-analysis. Int J Cardiol.

[26] Bajwa HZ, Do L, Suhail M, Hetts SW, Wilson MW, Saeed M (2014). MRI demonstrates a decrease in myocardial infarct healing and increase in compensatory ventricular hypertrophy following mechanical microvascular obstruction. J Magn Reson Imaging.

[27] Khan JN, Greenwood JP, Nazir SA, Lai FY, Dalby M, Curzen N, Hetherington S, Kelly DJ, Blackman D, Peebles C, Wong J, Flather M, Swanton H, Gershlick AH, McCann GP (2016). Infarct Size Following Treatment With Second- Versus Third-Generation P2Y12 Antagonists in Patients With Multivessel Coronary Disease at ST-Segment Elevation Myocardial Infarction in the CvLPRIT Study. J Am Heart Assoc.

[28] O’Donoghue ML, Morrow DA, Cannon CP, Jarolim P, Desai NR, Sherwood MW, Murphy SA, Gerszten RE, Sabatine MS (2016). Multimarker Risk Stratification in Patients With Acute Myocardial Infarction. J Am Heart Assoc.

[29] De Luca G, Van’t Hof AW, de Boer MJ, Ottervanger JP, Hoorntje JC, Gosselink AT, Dambrink JH, Zijlstra F, Suryapranata H (2004). Time-to-treatment significantly affects the extent of ST-segment resolution and myocardial blush in patients with acute myocardial infarction treated by primary angioplasty. Eur Heart J.

[30] de Waha S, Desch S, Eitel I, Fuernau G, Lurz P, Leuschner A, Grothoff M, Gutberlet M, Schuler G, Thiele H (2012). Relationship and prognostic value of microvascular obstruction and infarct size in ST-elevation myocardial infarction as visualized by magnetic resonance imaging. Clin Res Cardiol.

[31] Hamirani YS, Wong A, Kramer CM, Salerno M (2014). Effect of microvascular obstruction and intramyocardial hemorrhage by CMR on LV remodeling and outcomes after myocardial infarction: a systematic review and meta-analysis. JACC Cardiovasc Imaging.

[32] Lombardo A, Niccoli G, Natale L, Bernardini A, Cosentino N, Bonomo L, Crea F (2012). Impact of microvascular obstruction and infarct size on left ventricular remodeling in reperfused myocardial infarction: a contrast-enhanced cardiac magnetic resonance imaging study. Int J Cardiovasc Imaging.

[33] Neizel M, Futterer S, Steen H, Giannitsis E, Reinhardt L, Lossnitzer D, Lehrke S, Jaffe AS, Katus HA (2009). Predicting microvascular obstruction with cardiac troponin T after acute myocardial infarction: a correlative study with contrast-enhanced magnetic resonance imaging. Clin Res Cardiol.

[34] Younger JF, Plein S, Barth J, Ridgway JP, Ball SG, Greenwood JP (2007). Troponin-I concentration 72 h after myocardial infarction correlates with infarct size and presence of microvascular obstruction. Heart.

[35] Reichlin T, Hochholzer W, Bassetti S, Steuer S, Stelzig C, Hartwiger S, Biedert S, Schaub N, Buerge C, Potocki M, Noveanu M, Breidthardt T, Twerenbold R, Winkler K, Bingisser R, Mueller C (2009). Early diagnosis of myocardial infarction with sensitive cardiac troponin assays. N Engl J Med.

[36] Feistritzer HJ, Klug G, Reinstadler SJ, Reindl M, Mayr A, Mair J, Metzler B (2016). Novel biomarkers predicting cardiac function after acute myocardial infarction. Br Med Bull.

[37] George M, Shanmugam E, Srivatsan V, Vasanth K, Ramraj B, Rajaram M, Jena A, Sridhar A, Chaudhury M, Kaliappan I (2015). Value of pentraxin-3 and galectin-3 in acute coronary syndrome: a short-term prospective cohort study. Ther Adv Cardiovasc Dis.

[38] Iwakura K, Ito H, Kawano S, Okamura A, Kurotobi T, Date M, Inoue K, Fujii K (2006). Chronic pre-treatment of statins is associated with the reduction of the no-reflow phenomenon in the patients with reperfused acute myocardial infarction. Eur Heart J.

[39] Reinstadler SJ, Stiermaier T, Fuernau G, de Waha S, Desch S, Metzler B, Thiele H, Eitel I (2016). The challenges and impact of microvascular injury in ST-elevation myocardial infarction. Expert Rev Cardiovasc Ther.

[40] George M, Ganesh MR, Sridhar A, Jena A, Rajaram M, Shanmugam E, Dhandapani VE (2015). Evaluation of Endothelial and Platelet Derived Microparticles in Patients with Acute Coronary Syndrome. J Clin Diagn Res.

[41] Karim MA, Majumder AA, Islam KQ, Alam MB, Paul ML, Islam MS, Chowdhury KN, Islam SM (2015). Risk factors and in-hospital outcome of acute ST segment elevation myocardial infarction in young Bangladeshi adults. BMC Cardiovasc Disord.

[42] Payne AR, Berry C, Doolin O, McEntegart M, Petrie MC, Lindsay MM, Hood S, Carrick D, Tzemos N, Weale P, McComb C, Foster J, Ford I, Oldroyd KG (2012). Microvascular Resistance Predicts Myocardial Salvage and Infarct Characteristics in ST-Elevation Myocardial Infarction. J Am Heart Assoc.

